# Changes in choroidal circulation hemodynamics during the menstrual cycle in young, healthy women

**DOI:** 10.1371/journal.pone.0270501

**Published:** 2022-06-27

**Authors:** Mayumi Haneda, Yuki Hashimoto, Airi Mishima, Daichi Saito, Takeshi Yoshitomi

**Affiliations:** Department of Orthoptics, Faculty of Medicine, Fukuoka International University of Health and Welfare, Fukuoka, Japan; Icahn School of Medicine at Mount Sinai, UNITED STATES

## Abstract

**Purpose:**

The current study aimed to investigate the time course of changes in choroidal circulation hemodynamics and their relationship to systemic circulation dynamics during the normal menstrual cycle in young, healthy women using laser speckle flowgraphy (LSFG).

**Methods:**

This prospective study included 26 eyes from 13 young, healthy women (21.3 ± 4.0 years) with a normal menstrual cycle and 24 eyes from 12 young, healthy men (21.8 ± 4.4 years) as a control group. The macular mean blur rate (MBR), a quantitative index of relative blood flow velocity in the choroid, was measured using LSFG. MBR, intraocular pressure (IOP), systolic blood pressure (SBP), diastolic blood pressure (DBP), mean blood pressure (MBP), and ocular perfusion pressure (OPP) were evaluated in the late follicular phase and mid-luteal phase in women and at baseline and 10 days after baseline in men, respectively.

**Results:**

In the female group, IOP, SBP, DBP, MBP, and OPP values were significantly higher in the mid-luteal phase than those observed in the late follicular phase (*P* = 0.035, *P* < 0.001, *P* = 0.041, *P* = 0.001, *P* = 0.014, respectively). The average macular MBR values in the late follicular phase and mid-luteal phase were 12.7 ± 5.3 and 13.7 ± 6.6 (+7.7 ± 19.4%), representing a significant increase in the mid-luteal phase (*P* = 0.041). The rate of change in MBR exhibited a significant positive correlation with changes in DBP and MBP (*R* = 0.456, *P* = 0.019 and *R* = 0.474, *P* = 0.014). However, there were no significant changes in any of the factors in the male group during the study period.

**Conclusions:**

Our results suggest that in young, healthy women with a normal menstrual cycle, choroidal blood flow velocity decreases during the late follicular phase and increases during the mid-luteal phase, depending on systemic circulatory dynamics.

## Introduction

Normal menstruation comprises a cycle of four phases (menstrual, follicular, ovulatory, and luteal), with the entire cycle lasting approximately 28 days from the start of menstruation. In the follicular phase, estradiol is produced in the granulosa cells of the follicle, which is involved in the development of the endometrium [1, 2]. Endometrial thickness has been reported to increase during the follicular phase of the menstrual cycle, peak prior to ovulation, plateau during the early luteal phase, and subsequently decline prior to menstruation [3, 4]. Additional research suggests that the volume of the dominant ovary increases during the follicular phase, decreases after follicular rupture, and increases again during the luteal phase [5]. In terms of hormonal changes during the menstrual cycle, estrogen levels are high while progesterone levels are low during the late follicular phase. In contrast, levels of both estrogen and progesterone are high during the mid-luteal phase [6].

Blood pressure (BP) increases during the luteal phase [7], a phenomenon related to an increase in sympathetic nervous system activity [8–10]. This change in BP during the luteal phase is also affected by an increase in progesterone [11, 12]. Previous studies have noted that the endometrial vasculature changes significantly during the menstrual cycle, peaking approximately 3 days prior to ovulation and reaching its lowest point at 5 days post-ovulatory descent before and after implantation [13]. A serial transvaginal ultrasonography study of the normal menstrual cycle reported that the peak velocity of systolic blood flow increases significantly in the dominant follicle and ovarian stroma, and that the pulsatility index of the uterine artery on the side of the developing follicle decreases during the mid-luteal phase, becoming significantly lower than that on the contralateral side [14]. Additionally, cerebral blood flow values of the frontal pole increase during the follicular phase compared to those in the luteal phase [15].

Laser speckle flowgraphy (LSFG) is primarily used to evaluate the velocity of ocular blood flow [16–20]. In particular, the macula is considered to be largely affected by choroidal circulation hemodynamics because it is in the foveal avascular zone. The mean blur rate (MBR), automatically calculated from variations in the degree of blurring, is a quantitative index of the relative blood flow velocity. Therefore, LSFG is suitable for observing changes in choroidal hemodynamics in patients with various chorioretinal diseases [16, 18–20]. In addition, although a study suggests that there are no sex-related differences in choroidal MBR in normal eyes, changes in MBR over time in men and women remain unclear [21].

In central serous chorioretinopathy (CSC), the pathogenesis of which involves sympathetic hyperactivity, the MBR is increased in the acute stage [18, 19]. Similar findings are observed in patients with hypertensive choroidopathy (HTC), in which severe hypertension causes changes in the retinal pigment epithelium and choroid [20]. While LSFG is useful for investigating choroidal circulatory dynamics caused by sympathetic and systemic circulatory changes, the relationship between changes in choroidal hemodynamics and systemic circulatory dynamics during the normal menstrual cycle remains unknown.

The current study aimed to investigate the time course of changes in choroidal circulation hemodynamics and their relationship to systemic circulation dynamics during the normal menstrual cycle in young, healthy women, using LSFG.

## Materials and methods

### Participants

This study was approved by the ethics committee of Fukuoka International University of Health and Welfare (approval ID: 20-fiuhw-023) and adhered to the tenets of the Declaration of Helsinki. Written informed consent was obtained from all participants after the nature and possible consequences of the study had been explained.

This prospective study included 26 eyes from 13 young, healthy women with a normal menstrual cycle and 24 eyes from 12 young, healthy men as a control group. The number of participants in this study was based on previous reports describing the circulatory dynamics and sympathetic activity associated with the normal menstrual cycle [5, 6, 8, 10, 14, 15]. Post-hoc tests showed that our sample size was almost sufficient, yielding a statistical power of 78.2% (1 − β = 0.782). All participants had a best-corrected visual acuity (BCVA) ≥20/20, and only those with no ophthalmic or cardiovascular disease and no systemic or ophthalmic drug use were enrolled. In addition, for female participants, normal menstruation was defined as a menstrual cycle of 28 days (range: 25 to 30 days) [22]. Participants in the female group underwent assessments in the late follicular phase (day 13 ± 2 days) and the mid-luteal phase (day 23 ± 3 days), while those in the male group underwent assessments at baseline and 10 days after baseline.

Each participant underwent thorough examinations including BCVA, slit-lamp microscopy, and color fundus photography; additionally, intraocular pressure (IOP), systolic blood pressure (SBP), diastolic blood pressure (DBP), and LSFG were measured.

### LSFG measurement

**Fig 1:**
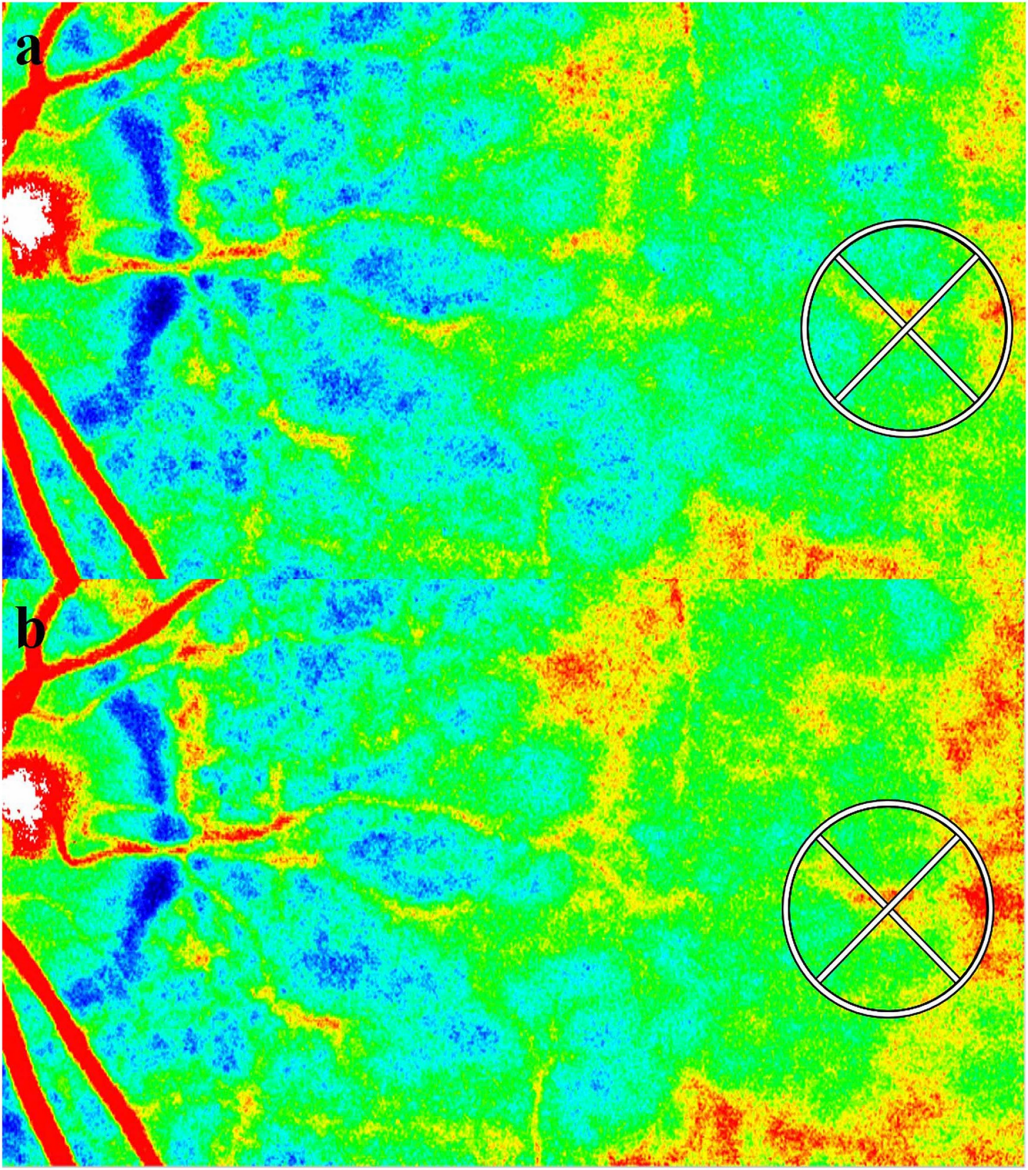
Composite color map images of the mean blur rate (MBR) measured using laser speckle flowgraphy in a representative participant (case 8). Blue coloring indicates low MBR, while red coloring indicates high MBR. MBR within the circle in the mid-luteal phase (b) increased by 19.8% compared to that in the late follicular phase (a).

LSFG-NAVI (Softcare Ltd, Fukuoka, Japan) targets moving red blood cells in the eye using an 830 nm diode laser to illuminate the ocular fundus. The laser speckle method has the advantage of allowing for non-invasive, quantitative, repeated examinations and reproducible results [16, 17, 23, 24]. Measurement using this system requires approximately 4 seconds. In this study, three consecutive LSFG measurements were obtained at each of the two evaluation points (late follicular phase and the mid-luteal phase for women, baseline and 10 days after baseline for men) to evaluate changes in choroidal blood flow velocity at the macula (Fig 1), excluding the large retinal vessels. At the follow-up assessment, each circle was automatically set using LSFG Analyzer software (v 3.0.47; Softcare Ltd., Fukuoka, Japan) at the same site where the circle was set at baseline. Changes in the average MBR were determined based on the rates of change in the average MBR against the initial baseline values (defined as 100%) [18–20]. LSFG measurements were obtained by three authors (M.H., Y.H., and A.M.).

### IOP, BP, and ocular perfusion pressure

For all participants, IOP and BP were measured at each evaluation point. Within a certain range, the relationship between choroidal blood flow and ocular perfusion pressure (OPP) is linear [25]. OPP was calculated from IOP and BP. Mean blood pressure (MBP) and OPP were calculated from IOP, SBP and DBP according to the following equations:

$$MBP=DBP\;+\;\;1/3\left(SBP\;-\;DBP\right)$$ (1)

OPP=2/3MBP–IOP (2)

### Statistics

All results are expressed as the mean ± standard deviation (SD). The Wilcoxon signed-rank test was used to examine changes in IOP, SBP, DBP, MBP, OPP, and MBR values. Spearman’s rank correlation test was used to determine the relationships between the rate of change in MBR and other factors including IOP, SBP, DBP, MBP, and OPP. For all tests, *P* values less than 0.05 were considered statistically significant. Cohen’s d measure was used as a post hoc analysis, to estimate the study’s statistical power based on our sample size.

## Results

The demographic characteristics of all participants are shown in Tables 1 and 2. Thirteen young, healthy women (mean age: 21.3 ± 4.0 years) and 12 young, healthy men (mean age: 21.8 ± 4.4 years) were included. There were no significant differences in the mean age of the two groups (*P* = 0.421).

**Table 1:** Characteristics of and changes in intraocular pressure and systemic factors in female participants.

Case	Age	Eye	IOP (mmHg)		SBP (mmHg)		DBP (mmHg)		MBP (mmHg)		OPP (mmHg)		MBR	
			Late FP	Mid-LP	Late FP	Mid-LP	Late FP	Mid-LP	Late FP	Mid-LP	Late FP	Mid-LP	Late FP	Mid-LP
1	20	R	10.0	9.3	111.5	117.5	68.0	74.0	82.5	88.5	45.0	49.7	7.8	8.5
		L	10.3	9.0							44.7	50.0	7.9	8.3
2	20	R	12.7	13.0	107.5	107.5	72.5	70.5	84.2	82.8	43.4	42.2	14.7	14.3
		L	15.0	14.0							41.1	41.2	13.2	12.1
3	20	R	14.0	15.7	103.0	115.5	68.5	69.0	80.0	84.5	39.3	40.6	23.3	26.5
		L	13.0	17.7							40.3	38.6	20.9	25.0
4	21	R	10.0	11.0	98.0	109.0	60.0	58.5	72.7	75.3	38.4	39.2	10.6	10.8
		L	11.3	10.7							37.1	39.5	9.3	7.6
5	20	R	10.3	12.7	102.5	113.0	69.5	79.5	80.5	90.7	43.4	47.7	25.2	32.9
		L	10.3	17.7							43.4	42.7	24.3	24.0
6	20	R	17.7	15.0	112.0	130.5	70.5	102.5	84.3	111.8	38.5	59.6	11.3	12.4
		L	15.7	15.0							40.5	59.6	11.1	11.5
7	19	R	10.0	13.0	102.5	112.0	67.5	74.5	79.2	87.0	42.8	45.0	10.7	15.9
		L	11.7	14.7							41.1	43.3	8.1	11.5
8	35	R	10.3	12.0	89.6	101.0	59.5	64.5	69.5	76.7	36.1	39.1	15.0	17.9
		L	10.7	12.0							35.7	39.1	11.1	13.3
9	20	R	17.3	16.0	107.5	105.5	67.5	73.0	80.8	83.8	36.6	39.9	16.4	15.3
		L	16.3	17.0							37.6	38.9	13.6	15.0
10	20	R	11.0	11.0	96.0	97.0	57.0	56.5	70.0	70.0	35.7	35.7	6.3	9.4
		L	11.0	11.0							35.7	35.7	7.1	7.2
11	20	R	9.0	11.0	85.5	93.0	54.0	64.5	74.0	74.0	34.0	38.3	14.3	13.9
		L	10.0	8.7							33.0	40.6	11.6	12.9
12	21	R	12.0	15.7	106.5	121.0	71.0	70.5	87.3	87.3	43.2	42.5	7.2	7.3
		L	12.7	15.7							42.5	42.5	7.5	7.6
13	21	R	11.7	12.3	103.0	102.5	70.5	58.0	81.3	72.8	42.5	36.3	12.3	8.9
		L	12.0	12.0							42.2	36.6	8.8	6.3
Mean ± SD	21.3 ± 4.0		12.2 ± 2.4	13.2 ± 2.6	101.9 ± 7.6	109.6 ± 9.9	65.8 ± 5.8	70.4 ± 11.5	77.9 ± 6.2	83.5 ± 10.4	39.8 ± 3.4	42.5 ± 6.2	12.7 ± 5.3	13.7 ± 6.6

SD, standard deviation; IOP, intraocular pressure; FP, follicular phase; LP, luteal phase; SBP, systolic blood pressure; DBP, diastolic blood pressure; MBP, mean blood pressure; OPP, ocular perfusion pressure; MBR, mean blur rate

**Table 2:** Characteristics of and changes in intraocular pressure and systemic factors in male participants.

Case	Age	Eye	IOP (mmHg)		SBP (mmHg)		DBP (mmHg)		MBP (mmHg)		OPP (mmHg)		MBR	
			Base-line	10 days	Base-line	10 days	Base-line	10 days	Base line	10 days	Base line	10 days	Base-line	10 days
1	20	R	12.3	11.3	119.0	119.0	73.0	69.0	88.3	85.7	46.6	45.8	17.0	18.5
		L	11.7	11.0							47.2	46.1	14.3	12.7
2	23	R	10.3	11.0	130.0	109.5	74.0	70.5	92.7	83.5	51.5	44.7	19.0	18.5
		L	9.3	12.0							52.5	43.7	11.3	10.5
3	21	R	8.3	8.7	123.5	110.5	71.5	72.0	88.8	84.8	50.9	47.9	8.6	9.4
		L	9.7	9.0							49.5	47.5	6.1	6.1
4	20	R	10.3	11.0	111.0	109.5	72.0	68.5	85.0	82.2	46.4	43.8	9.8	9.5
		L	10.7	11.3							46.0	43.5	10.6	9.9
5	20	R	10.3	12.3	112.0	118.5	73.5	73.0	86.3	88.2	47.3	46.5	9.3	9.8
		L	12.0	12.0							45.5	46.8	9.2	9.2
6	20	R	16.0	15.0	115.5	122.5	82.0	90.5	93.2	101.2	46.1	52.4	17.4	16.1
		L	12.7	13.0							49.4	54.5	16.9	15.1
7	36	R	13.0	12.7	114.0	110.5	66.0	77.0	82.0	88,2	41.7	46.1	11.2	10.3
		L	12.0	12.3							42.7	46.5	8.5	9.3
8	21	R	16.7	11.7	106.5	104.5	80.5	71.5	89.2	82.5	42.7	43.3	10.8	10.8
		L	15.7	13.0							43.8	42.0	9.0	8.3
9	21	R	11.0	12.0	117.0	112.5	76.5	69.0	90.0	83.5	49.0	43.7	7.0	8.0
		L	11.0	11.7							49.0	44.0	10.0	11.5
10	20	R	12.0	11.7	116.0	123.5	69.5	76.5	100.5	107.9	55.0	60.1	11.2	9.1
		L	11.3	11.3							55.7	60.5	9.6	10.0
11	20	R	11.7	12.0	124.0	105.5	76.0	65.5	92.0	78.8	49.6	40.6	14.6	15.2
		L	12.0	13.0							49.3	39.5	12.7	11.0
12	20	R	13.7	15.3	112.5	102.0	53.5	65.5	92.8	89.8	48.1	44.6	22.4	22.9
		L	13.7	16.0							48.1	43.9	10.6	14.0
Mean ± SD	21.8 ± 4.4		12.0 ± 2.0	12.1 ± 1.6	116.8 ± 6.2	112.3 ± 6.8	72.3 ± 7.1	72.4 ± 6.5	90.1 ± 4.5	88.0 ± 8.0	48.1 ± 3.5	46.6 ± 5.2	12.0 ± 4.0	11.9 ± 3.9

SD, standard deviation; IOP, intraocular pressure; SBP, systolic blood pressure; DBP, diastolic blood pressure; MBP, mean blood pressure; OPP, ocular perfusion pressure; MBR, mean blur rate

### IOP, BP, and OPP data

Changes in IOP and systemic factors are shown in Table 1 for women and Table 2 for men. In the female group, IOP, SBP, DBP, MBP, and OPP significantly increased in the late follicular phase and mid-luteal phase (*P* = 0.035, *P* < 0.001, *P* = 0.041, *P* = 0.001, and *P* = 0.014) (Table 3). Conversely, there were no significant changes in any factors in the male group during the study period (*P* = 0.346, *P* = 0.182, *P* = 0.969, *P* = 0.308, and *P* = 0.166) (Table 3).

**Table 3:** Comparison of intraocular pressure and systemic factors between female participants in the late follicular phase and mid- luteal phase and male participants at baseline and 10 days.

Female group (13 cases, 26 eyes)	Late follicular phase	Mid-luteal phase	*P* value
IOP (mmHg)	12.2 ± 2.4	13.2 ± 2.6	0.035
SBP (mmHg)	101.9 ± 7.6	109.6 ± 9.9	<0.001
DBP (mmHg)	65.8 ± 5.8	70.4 ± 11.5	0.041
MBP (mmHg)	77.9 ± 6.2	83.5 ± 10.4	0.001
OPP (mmHg)	39.8 ± 3.4	42.5 ± 6.2	0.014
MBR (%)	100.0 ± 0.0	107.7 ± 19.4	0.041
Male group (12 cases, 24 eyes)	Baseline	10 days	*P* value
IOP (mmHg)	12.0 ± 2.0	12.1 ± 1.6	0.346
SBP (mmHg)	116.8 ± 6.2	112.3 ± 6.8	0.182
DBP (mmHg)	72.3 ± 7.1	72.4 ± 6.5	0.969
MBP (mmHg)	90.1 ± 4.5	88.0 ± 8.0	0.308
OPP (mmHg)	48.1 ± 3.5	46.6 ± 5.2	0.166
MBR (%)	100.0 ± 0.0	100.3 ± 10.9	0.931

IOP, intraocular pressure; SBP, systolic blood pressure; DBP, diastolic blood pressure; MBP, mean blood pressure; OPP, ocular perfusion pressure; MBR, mean blur rate

Wilcoxon signed-rank test

### LSFG data

Changes in MBR are shown in Tables 1 and 2. In female participants, the average macular MBR values in the late follicular phase and mid-luteal phase were 12.7±5.3 and 13.7±6.6, respectively, while these values were 12.0±4.0 and 11.9±3.9 in male participants, respectively. The macular MBR values significantly increased by 7.7±19.4% in the late follicular phase in female participants (*P* = 0.041), but there was no significant difference at 10 days after baseline in male participants (*P* = 0.931) (Table 3).

### Correlation between MBR and other factors

**Fig 2:**
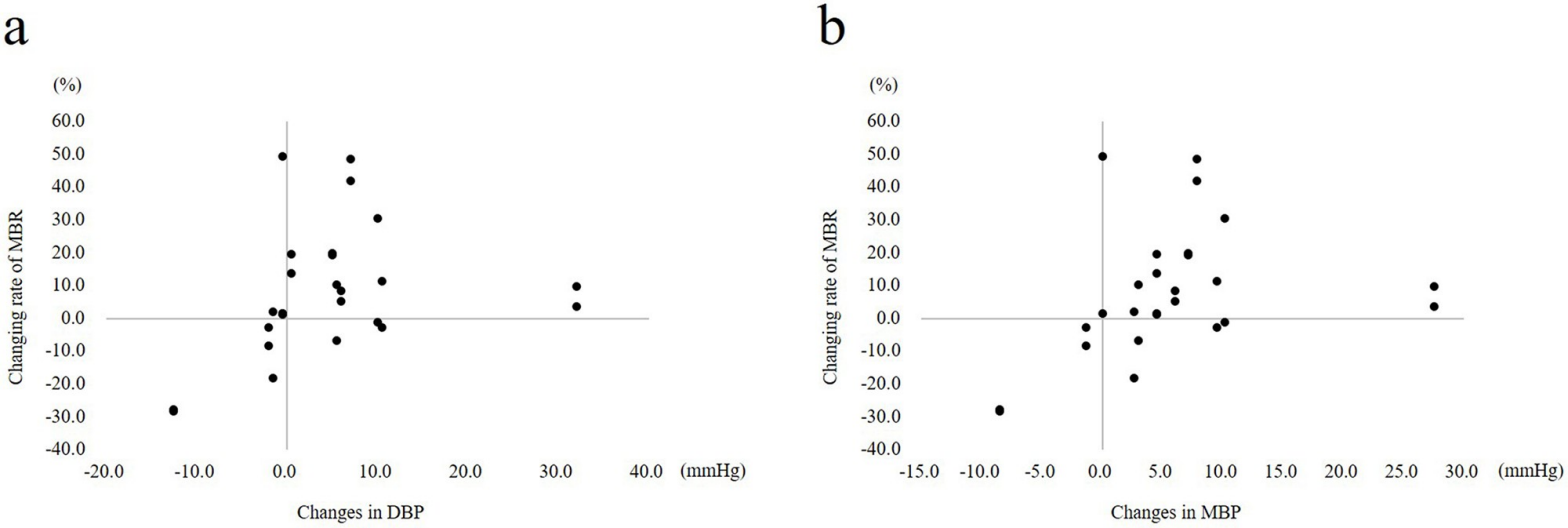
Correlations between the rate of change in macular MBR and mean diastolic blood pressure (DBP) or mean blood pressure (MBP) in the female group. (a) The MBR increase exhibited a positive correlation with the DBP increase in the late follicular phase and mid-luteal phase (*R* = 0.456, *P* = 0.019). (b) There was a statistically significant positive correlation between the MBR increase and the MBP increase in the late follicular phase and mid-luteal phase (*R* = 0.474, *P* = 0.014).

The factors significantly correlated with the rate of change in MBR between the follicular phase and mid-luteal phase are shown in Fig 2. The rate of change in MBR exhibited a significant positive correlation with changes in DBP and MBP (Spearman’s rank correlation test, *R* = 0.456, *P* = 0.019 and *R* = 0.474, *P* = 0.014, respectively). However, no statistically significant correlation was observed between MBR and other factors including IOP, SBP, and OPP (Spearman’s rank correlation test, *R* = 0.261, *P* = 0.199; *R* = 0.318, *P* = 0.113; and *R* = 0.328, *P* = 0.102, respectively).

## Discussion

To the best of our knowledge, the present study is the first to investigate the course of quantitative changes in choroidal blood flow velocity during the normal menstrual cycle in young, healthy women. Significant increases in MBR values at the macula were associated with increases in systemic circulation factors including SBP, DBP, MBP, and OPP. Furthermore, the rate of change in the macular MBR was positively correlated with the changes in DBP and MBP. In the normal menstrual cycle, sympathetic hyperactivity during the luteal phase suggests that choroidal blood flow velocity increases along with increases in systemic circulatory dynamics. In contrast, no significant changes in any factors were observed between baseline and 10 days later in the male control group.

Autonomic nervous system activity is known to vary during the menstrual cycle. In young women, sympathetic activity increases during the luteal phase, while parasympathetic activity decreases [8–10]. In the late follicular phase, estrogen levels are high and progesterone levels are low, while levels of both are higher in the mid-luteal phase [6]. These changes are caused by fluctuations in ovarian hormones, such as estrogen and progesterone, each of which influences the autonomic nervous system. In particular, increased progesterone has been shown to increase BP [11, 12]. Therefore, these mechanisms lead to an increase in systemic circulatory dynamics, including BP, during the luteal phase [6–12]. The results of this study, in which BP increased during the luteal phase than that in the follicular phase, support findings described in a previous report [7].

Previous studies have reported that, in non-inflammatory diseases with an etiology characterized by sympathetic hyperactivity, such as CSC, choroidal blood flow velocity increases in the acute phase and decreases in the regression phase [18, 19]. There are two hypotheses that may explain the increase in macular MBR in eyes with acute CSC. First, vasoconstriction of choroidal arteries and secondary overflow into large choroidal vessels may occur due to activation of sympathetic α-adrenoceptors. Alternatively, the increase in cardiac output due to activation of sympathetic β-adrenoceptors may increase blood flow to the entire choroid [19]. Furthermore, changes in the MBR of HTC, the pathogenesis of which involves severe hypertension, are similar to those observed for CSC. Consequently, researchers have hypothesized that HTC is caused by hyperperfusion of the choroid [20]. In the present study, choroidal blood flow velocity increased during the mid-luteal phase, when sympathetic nervous system activity was high, and BP and OPP increased accordingly, indicating that these changes were consistent with the above hypotheses. However, contrasting results have also been published, suggesting that choroidal blood flow velocity decreases at the acute stage of Vogt–Koyanagi–Harada disease due to its inflammatory etiology [26, 27].

The primary limitations of this study were: first, the relatively small sample size and the resultant statistical power being minimally below the desired threshold (78.2% vs 80.0%); second, the absence of other choroidal examinations. To further investigate choroidal changes in healthy participants, the relationship between choroidal function and morphology should be assessed in large samples using both LSFG and optical coherence tomography angiography.

## Conclusions

Our results suggest that in young, healthy women with a normal menstrual cycle, choroidal blood flow velocity decreases during the late follicular phase and increases during the mid-luteal phase, depending on systemic circulatory dynamics. Thus, LSFG may be a useful method for non-invasively and quickly performing quantitative assessments of choroidal and systemic involvement during the normal menstrual cycle. In addition, the MBR was 7.7% higher in the mid-luteal phase than that in the late follicular phase, indicating that it may be necessary to consider the menstrual cycle in the interpretation of LSFG results in the future.
